# The volume of the subthalamic nucleus in spinocerebellar ataxia type 3: potential relevance for the clinical phenotype and treatment of parkinsonian symptoms with deep brain stimulation

**DOI:** 10.1007/s00415-024-12792-5

**Published:** 2024-12-12

**Authors:** Martina Minnerop, Peter Pieperhoff, Saskia Elben, Christian Johannes Hartmann, Tomke Müttel, Ulrike Kahlen, Ullrich Wüllner, Thomas Klockgether, Lars Wojtecki, Svenja Caspers, Katrin Amunts, Jan Vesper, Alfons Schnitzler, Stefan Jun Groiss

**Affiliations:** 1 Institute of Neuroscience and Medicine (INM-1), Research Center Jülich GmbH, Leo-Brandt-Str. 1, 52425 Jülich, Germany; 2 Institute of Clinical Neuroscience and Medical Psychology, Medical Faculty and University Hospital Düsseldorf, Heinrich Heine University Düsseldorf, Düsseldorf, Germany; 3 Department of Neurology, Center for Movement Disorders and Neuromodulation, Medical Faculty and University Hospital Düsseldorf, Heinrich Heine University Düsseldorf, Düsseldorf, Germany; 4 Department of Neurology, Medical Faculty and University Hospital Düsseldorf, Heinrich Heine University Düsseldorf, Düsseldorf, Germany; 5 Department of Neurology, Hospital Leverkusen, Leverkusen, Germany; 6 Department of Parkinson, Sleep and Movement Disorders, Centre of Neurology, University Hospital Bonn, Bonn, Germany; 7 German Center for Neurodegenerative Diseases (DZNE), Bonn, Germany; 8 Institute for Anatomy I, Medical Faculty and University Hospital Düsseldorf, Heinrich Heine University Düsseldorf, Düsseldorf, Germany; 9 C. and O. Vogt Institute for Brain Research, Medical Faculty and University Hospital Düsseldorf, Heinrich Heine University Düsseldorf, Düsseldorf, Germany; 10 Department of Functional and Stereotactic Neurosurgery, Center for Neuromodulation, Medical Faculty and University Hospital Düsseldorf, Heinrich Heine University Düsseldorf, Düsseldorf, Germany; 11 Neurocenter, Düsseldorf, Germany

**Keywords:** Parkinson, Ataxia, SCA3, DBS, STN, MRI

Dear Madam or Sir,

Spinocerebellar ataxia type 3 (SCA3) is an autosomal dominant inherited neurodegenerative disorder caused by cytosine–adenine–guanine (CAG) repeats resulting in an expanded polyglutamine tract within the ataxin-3 protein. Cerebellar atrophy and progressive ataxia are key features, but a variety of non-ataxia symptoms, including parkinsonian symptoms may occur as well [1].

Neuropathological as well as in vivo MRI-based morphometry studies demonstrated atrophy in nuclei and fiber tracts of the brainstem, cerebellum and basal ganglia [1]. The striatonigral system and in particular the subthalamic nucleus (STN) show severe atrophy and reduction of dopamine transporter (DAT) activity as much as in Parkinson’s disease (PD), but in SCA3 patients of European descent, parkinsonian symptoms are only rarely observed (rigidity in ca. 10%) and a putative masking by cerebellar symptoms is debated [2, 3]. Schöls et al*.* [4] described in their *post mortem* study a severe neuronal loss of the STN in all investigated SCA3 cases, except in a single SCA3 patient who showed parkinsonian features during lifetime. The authors concluded that the neuronal loss of the STN counteracts overt parkinsonian symptoms in SCA3, which would be expected because of the severe neurodegeneration of the dopaminergic substantia nigra. They further pointed out that the effect of severe neuronal loss of the STN in SCA3 might be somehow comparable to the therapeutic effects observed in deep brain stimulation (DBS) of the STN for PD [4].

Thus, the degree of atrophy of the STN may have an impact on the manifestation of parkinsonian symptoms in SCA3. As a consequence, DBS of the STN in SCA3 patients with parkinsonian symptoms (SCA3-PS) might have a beneficial effect only when sufficient vital tissue is still present in the target region regardless of the criterion of DOPA-responsiveness. Therefore, we examined the variability of the STN volume in a sample of eleven SCA3 patients, using MRI-based volumetry. This sample included a single SCA3-PS patient, who was successfully treated by bilateral STN-DBS with a long-lasting effect during a follow-up interval of 3 years.

Patients were compared with an age- and sex-matched control sample, and with a second extended control sample (*N* = 235) with elder participants of the 1000BRAINS study [6] (Table 1). Additionally, clinical details are reported regarding the single SCA3-PS patient. We hypothesized that the SCA-PS patient had a larger STN volume than most of the SCA3 patients without parkinsonian symptoms, and that it was closer to the normal range.

**Table 1:** Demographic, clinical and volume data

	Control group Mean ± SD [range]	Extended control group Mean ± SD [range]	SCA3 group Mean ± SD [range]	SCA3-PS
*Demographic and clinical data*				
N	11	224	11	1
Sex (m/f)	7/4	110/114	7/4	1/0
Age [years]	39.1 ± 8.3 [30.3–53.2]	64.1 ± 10.8 [26.2–84.2]	40.6 ± 9.4 [30.0–63.8]	38.1
CAG-Repeat length	–	–	72 ± 4 [63–77]	67
Age at disease onset [years]	–	–	33.8 ± 9.9 [24.3–55.2]	31.3
Disease duration [years]	–	–	6.7 ± 2.5 [2.6–10.3]	6.8
Ataxia severity (SARA score)	–	–	6.8 ± 6.0 [1.0–21.5]	6.5
Non-ataxia Signs (INAS score)	–	–	1.2 ± 1.3 [0–4]	2

ROI	Side				Group diff *p*_**uncorr**_-value	Group diff *p*_**FDR**_-value	Effect size (Cohen’s d)
*Volume data in mm*^*3*^* (mean ± SD)*							
STN	L	223.5 ± 21.9 [166.6–286.1]	187.5 ± 24.6 [142.6–222.9]	216.1	0.00012	0.00030	1.65
	R	237.8 ± 23.4 [179.0–294.5]	203.0 ± 25.7 [162.0–241.5]	229.9	0.0016	0.00320	1.49
	L + R	461.2 ± 44.4 [345.9–575.1]	390.5 ± 49.5 [307.3–464.4]	446.0	0.00036	0.000081	1.60
SNpc	L	186.6 ± 19.2 [136.8–238.1]	158.7 ± 21.0 [116.7–186.5]	181.6	< 0.00001	0.00001	1.46
	R	199.6 ± 19.9 [149.7–256.8]	165.5 ± 20.6 [134.4–201.8]	185.8	0.00001	0.00004	1.73
	L + R	386.2 ± 38.7 [288.9–486.2]	324.2 ± 41.0 [251.0–388.3]	367.5	0.00001	0.00002	1.61
Putamen	L	5913.3 ± 581.3 [4099.3–7385.7]	5736.2 ± 528.0 [4914.7–6387.8]	6164.6	0.10074	0,12,088	0.31
	R	6452.7 ± 598.1 [4796.1–8108.3]	6235.9 ± 660.1 [5227.4–7223.0]	6768.0	0.14463	0,14,463	0.36
	L + R	12,366.1 ± 1157.1 [8895.4–15,493.9]	11,927.1 ± 1176.1 [10142.1–13,610.8]	12,932.7	0.11433	0.12863	0.34
Caudate Nucleus	L	4057.8 ± 646.9 [2982.9–7925.1]	3719.1 ± 222.6 [3370.0–4073.4]	3506.6	0.13349	0.14134	0.54
	R	5351.7 ± 563.0 [4153.6–6979.2]	5117.3 ± 293.1 [4646.7–5607.5]	5083.0	0.08965	0.12088	0.43
	L + R	9409.5 ± 1120.2 [7231.8–13,851.5]	8836.5 ± 487.7 [8113.4–9602.3]	8589.6	0.09431	0.12088	0.53
Cerebellum	L	64,060.0 ± 6180.1 [48,993.1–81,855.6]	62,321.9 ± 3250.1 [56,726.0–68,791.8]	65,023.0	0.04817	0.08670	0.29
	R	63,923.4 ± 6388.9 [47,070.5–84,543.3]	60,703.0 ± 3970.1 [55,046.0–69,549.0]	62,820.9	0.09839	0.12088	0.52
	L + R	127,983.4 ± 12,420.5 [96,063.6–166,205.0]	123,024.9 ± 7174.3 [111,772.0–138,341.0]	127,843.9	0.06713	0.10986	0.41
Dentate Nucleus	L	1163.5 ± 127.6 [874.0–1622.5]	956.6 ± 170.5 [701.6–1149.7]	1086.1	< 0.00001	< 0.00001	1.16
	R	800.5 ± 93.8 [557.7–1130.0]	549.5 ± 97.7 [394.0–694.1]	642.9	< 0.00001	< 0.00001	2.37
	L + R	1964.0 ± 215.4 [1431.7–2752.5]	1506.1 ± 244.4 [1095.7–1841.7]	1728.9	< 0.00001	< 0.00001	1.75

*m* male, *f* female, *INAS* Inventory of Non-Ataxia Signs, *L* left, *p*_*FDR*_ p-value corrected for multiple comparison (False Discovery Rate), *p*_*uncorr*_ p-value uncorrected for multiple comparison, *R* right, *SARA* Scale for the Assessment and Rating of Ataxia, *SNpc* Substantia nigra pars compacta, *STN* subthalamic nucleus, *ROI* region of interest

According to the Inventory of Non-Ataxia Signs (INAS) [5], 6/11 SCA3 patients had between one to four different non-ataxia signs: (spasticity (*N* = 3), hyperreflexia (*N* = 1), fasciculations (*N* = 1), areflexia (*N* = 1), sensory symptoms (*N* = 3), brainstem oculomotor signs (*N* = 1), dystonia (*N* = 1), rigidity (*N* = 1), resting tremor (*N* = 1) (Table 1).

The single SCA3-PS received DAT imaging with [^123^I]FP-CIT-SPECT before DBS. Clinical effects of DBS in this SCA3-PS patient over three years were documented by applying several scores and questionnaires including the *Unified Parkinson's Disease Rating Scale* (UPDRS III), the *Ardouin Scale of Behavior in Parkinson's Disease (ABS)* and *Parkinson's Disease Questionnaire – 39* (PDQ-39). Cognitive performance was assessed with the *Montreal Cognitive Assessment* (MoCA). The study was approved by the respective Institutional Review Boards. Written informed consent was obtained from all participants.

MR images were acquired with a T1-weighted 3D MP-RAGE sequence, using a Siemens Sonata 1.5 T Scanner for the eleven SCA3 patients and matched controls (repetition time 2200 ms, echo time 3.93 ms, voxel size 1 × 1 × 1 mm^3^), and a Siemens Trio 3.0 T Scanner for the extended control sample (repetition time 2250 ms, echo time 3.03 ms, voxel size 1 × 1 × 1 mm^3^). MR images were visually inspected for imaging artifacts or brain structural abnormalities. This included also the inspection of T2/FLAIR images, which were part of the MRI protocol, but not further used in the present study. All images included in the analysis showed no artifacts or abnormalities. The single SCA3-PS patient and one control subject were scanned three times over 12 years, both with similar time intervals in between. All scans of the SCA3-PS patient were acquired before DBS electrode implantation. Region volumes were measured using Deformation-based morphometry [7]: Based on a high-dimensional nonlinear approach, individual MR images were registered to the reference brain of the Julich Brain and the AAL3 atlas and voxel-wise local volume ratio (LVR) maps were calculated. The individual LVRs were regionally summed based on the cytoarchitectonic maps not only of the STN, but also of the dentate nucleus, both part of the Julich-Brain atlas (https://julich-brain-atlas.de, also available via EBRAINS: https://ebrains.eu/) [8–10], and the maps of the total cerebellum, substantia nigra pars compacta (SNpc), putamen and caudate nucleus as part of the AAL3 atlas [11]. Group differences of each region volume were analyzed by an ANCOVA model with the categorical factors “group” (SCA3, Control) and “MR scanner” and the subjects’ age as covariates. Further, effect sizes (Cohen’s *d*) of group differences were calculated. Among patients, Spearman correlations of the age-adjusted region volumes (given by the residual volumes of the ANCOVA) with the CAG repeat length, disease duration and disease severity (ataxia score, SARA) were calculated. Age-dependent confidence and prediction bands for the region volumes within the control group were calculated by linear regression and visualized using SAS^®^ 9.4 (SAS Institute, Cary, USA).

At group level, SCA3 patients exhibited a pronounced reduction of the age-adjusted STN volume in comparison to both control samples (relative volume reduction of 13–17%, respectively, *p* < 0.0016, *d* = 1.60). STN volumes showed bilaterally a negative Spearman correlation with CAG repeat length (left/right: *ρ* = − 0.84/− 0.77, *p* = 0.0014/0.0055) and left-sided with disease severity (SARA score, *ρ* = − 0.68, *p* = 0.0208). Volume data of all regions analyzed are given in Table 1.

The SCA3-PS patient developed ataxia at age 31 and l-DOPA-responsive parkinsonian symptoms at age 35. He later suffered l-DOPA-related motor fluctuations with ON dyskinesia and OFF akinesia. Despite a l-DOPA, equivalent daily dose of 2961 mg at age 50 his mobility was satisfying for only 2.5 h/day. MRI showed at baseline a moderate cerebellar atrophy (Fig. 1c), and basal ganglia appeared normal (Fig. 1f). However, [^123^I]FP-CIT-SPECT revealed an almost complete bilateral loss of striatal DAT binding with only a residual uptake in the left caudate head (Fig. 1g). Estimation of the STN volume in the SCA3-PS patient excluded severe atrophy, supporting the clinical decision to perform STN-DBS using directional leads (Abbott SJM Infinity, Abbott, Texas, USA). Standard directional monopolar high-frequency stimulation (130 Hz, 60 µs and 1.5/2.0 mA for left/right STN) led to clear motor improvement (UPDRS III DBS off/medication off: 29, DBS on/medication off: 23, DBS on/medication on: 12). ON/OFF fluctuations disappeared and the daily levodopa dose was reduced by almost two-thirds to 1166 mg/day. These effects of DBS continued during the current follow-up period of 3 years—requiring only little adaptations of the stimulation parameters and the additional daily levodopa dose. This benefit was reflected by an improvement in the *ABS score* (8 vs 3 points) and in *PDQ-39 subscores* (e.g., ADL, pre-DBS 83.3 vs 79.2 at 3 years of follow-up). However, because of progressive cerebellar ataxia and polyneuropathy, the patient remained wheelchair-bound and suffered—as before DBS—from recurrent depressive episodes. Therefore, an anti-depressive drug treatment had to be re-initiated 2 years after DBS surgery. Cognition (MoCA score) remained stable.

**Fig. 1:**
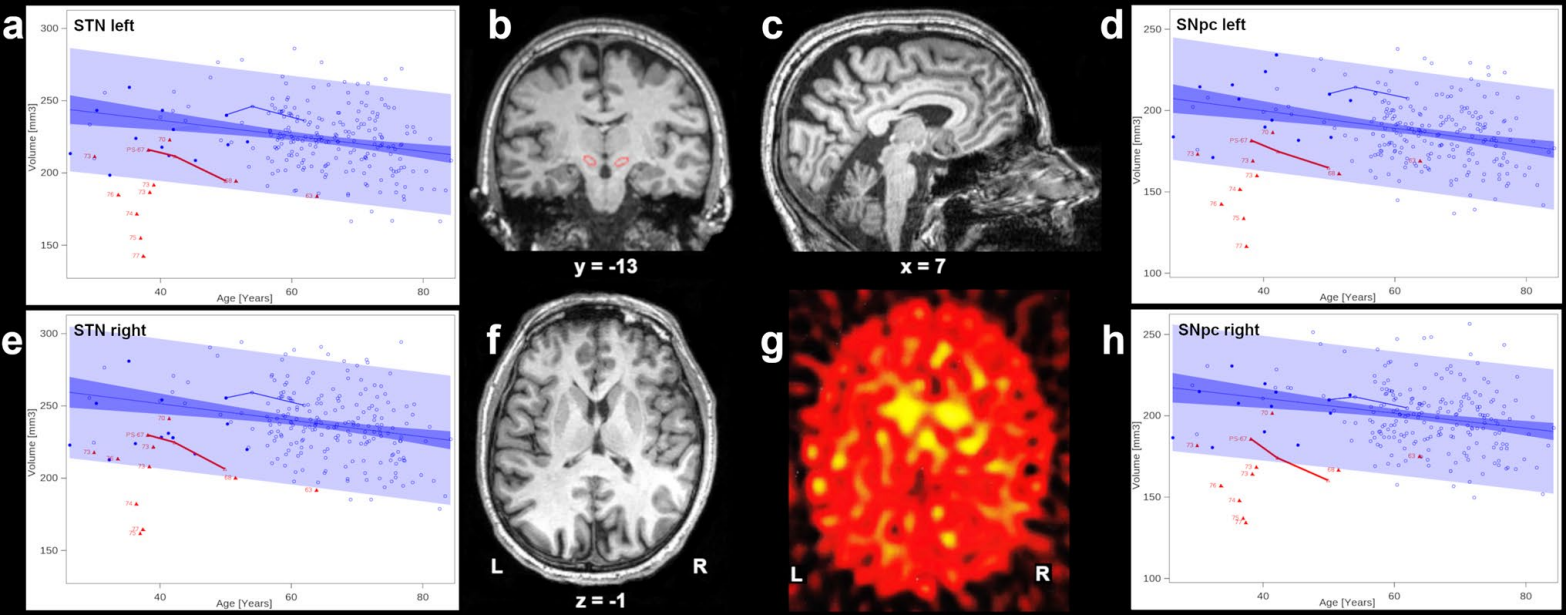
**b, c, f** 3 T MRI data of the SCA3 patient with parkinsonian symptoms at age 50, outlining in red the anatomical position of the right and left STN (**b**) demonstrating moderate cerebellar atrophy (**c**) and normal-appearing basal ganglia (**f**). **g** Dopamine transporter SPECT revealed bilateral loss of striatal uptake. **a**, **e**, **d**, **h** MRI-based volumes of the STN (**a**, **e**) and SNpc (**d**, **h**) in SCA3 patients (red, numbers indicate the individual repeat length of the expanded allele), age- and sex-matched healthy controls and elderly healthy participants of the 1000BRAINS study (blue). Filled circles and triangles refer to MRI data obtained at 1.5 T and non-filled circles and triangles to data obtained at 3 T. The blue area surrounding the age-dependent regression line represents the confidence interval of the age-dependent mean values of all control subjects. Longitudinal STN volume data of the single SCA3 patient with parkinsonian symptoms (red; data obtained 12 years and 8 years prior to DBS and shortly before DBS) and one healthy control at three different time points with similar intervals in between are connected by lines.

MR morphometry in SCA3 has revealed volume loss in particular of brainstem, cerebellum and striatum [12, 13] with most prominent volume change over time in caudate and putamen, followed by brainstem and cerebellum [14]. Neuropathologically, substantia nigra and the cerebellar dentate nucleus were demonstrated to undergo the most pronounced alterations [15]. Our in vivo volumetry data show in SCA3 patients a pronounced bilateral atrophy of dentate nucleus, SNpc and STN (Table 1, Fig. 1a, d, e, h), in line with literature [1, 12–15]. However, the only mild (non-significant) cerebellar and striatal atrophy might be due to the small sample size (i.e., statistically underpowered) or the specific profile of this cohort with comparable shorter disease duration. Despite a severe effect of the SNpc in almost all patients of our sample, parkinsonian symptoms were observed only in one with a comparable modest atrophy of the SNpc (Fig. 1d, h). Remarkably, the SNpc volume in our patient sample was about 17% below the controls’ volume, whereas a recent morphometry study reported a group difference between idiopathic Parkinson’s disease patients and controls of about 3.3% only [16]. Absence of parkinsonian symptoms in SCA3 and SCA2 patients in spite of severe atrophy of the striatonigral system and reduced DAT activity as in PD is well known but still not fully understood [3, 4]. Masking by cerebellar hypotonia is debated, but other neurological symptoms (neuropathy, dystonia) and mechanisms may also impede the detection of parkinsonian symptoms [17]. A common explanation for the occurrence of parkinsonian symptoms includes disinhibition of the STN as a result of inadequate dopaminergic neurotransmission. In SCA3 (and SCA2) patients, this could be attenuated by the severe atrophy of the STN: Schöls et al. suggested that pronounced atrophy of the STN in SCA3 could counteract parkinsonian symptoms resulting in an ataxia phenotype, while less STN atrophy might result in parkinsonian symptoms [4]. In line, the STN volume in our single SCA3-PS patient appeared to be closer to the normal range, with a mild decrease over time probably reflecting a mixture of disease- and age-related effects compared to controls (Fig. 1a, e). In addition, the patient showed a long-lasting benefit after STN-DBS and, as in PD patients, requiring some adjustment of DBS parameters during follow-up.

Nevertheless, the proposed association between the presence or absence of parkinsonian symptoms and STN volume should be confirmed in larger SCA3 samples with more patients presenting parkinsonian symptoms, covering a broader spectrum of CAG repeats and a long follow-up. Our single SCA3-PS patient, carried the second lowest CAG repeat length in our sample (67 repeats), similar to the case (69 repeats) described by Schöls et al. [4] and to a recently published case report of DBS in a SCA3 patient (66 repeats [18]). This fits to the observation that, inter alia, intermediate CAG repeat expansions are associated with parkinsonian symptoms in SCA2 and SCA3 [19]. However, less-reduced STN volumes and the observation of parkinsonian symptoms cannot be attributed solely to a later entry in the neurodegenerative process as might initially be assumed based on the known inverse association between the CAG repeat length and age at disease onset [1]: Three other patients with repeat lengths < 70 had also STN volumes close to the normal range, but an ataxia phenotype and no parkinsonian symptoms. While the repeat length seems to have an impact on the STN volume, the interaction with the motor phenotype seems thus less clear. Whether a decline of the STN volume over time—as observed in our patient—might limit the DBS benefit is currently unknown, but at least during the follow-up period of three years, the patient continued to benefit from DBS. Of note, mildly reduced MR volume of the (left) STN has also been described in PD patients, but did not correlate with motor scores [16].

In conclusion, based on the preserved STN in SCA3-PS in vivo and *post mortem* (our data, [4]) and the observed clinical effect in single cases ([18], our data) in combination with similar electrophysiological pattern in SCA-PS and PD [20], DBS seems to be in general justified and effective in SCA3-PS patients for the alleviation of parkinsonian symptoms. This predisposes SCA3 and eventually other SCA patients with parkinsonian symptoms (e.g., SCA2, SCA17) as potential candidates for DBS.

## Data Availability

Data are available from the corresponding author upon reasonable request by qualified academic investigators.
